# Usage and Attitudes of Physicians in Japan Concerning Traditional Japanese Medicine (Kampo Medicine): A Descriptive Evaluation of a Representative Questionnaire-Based Survey

**DOI:** 10.1155/2012/139818

**Published:** 2012-01-26

**Authors:** E. C. Moschik, C. Mercado, T. Yoshino, K. Matsuura, K. Watanabe

**Affiliations:** ^1^School of Dentistry, Medical University Graz, Mozartgasse 12/1, 8010 Graz, Austria; ^2^School of Medicine, Johns Hopkins University, 3400 N Charles Street, Baltimore, MD 21218, USA; ^3^Center for Kampo Medicine, Keio University School of Medicine, 35 Shinanomachi, Shinjuku-ku, Tokyo 160-8582, Japan

## Abstract

Kampo medicine has been the primary medical model in Japan until the mid 1800s, regained a prominent role in today's Japanese medical system. Today, 148 herbal Kampo formulas can be prescribed under the national health insurance system, allowing physicians to integrate Kampo in their daily practice. This article aims to provide information about the extent to which Kampo is now used in clinics throughout Japan and about physician's current attitudes toward Kampo. We used the results of a 2008 survey that was administered to physicians throughout Japan (*n* = 684). The data showed that 83.5% of physicians currently use Kampo in the clinic, although the distribution of physicians who use Kampo differ widely depending on the specialty and provided a breakdown of Kampo usage by specialty. It will be interesting to see how each specialty incorporates Kampo into its respective field as Kampo continues to play a pertinent role in Japanese medical system.

## 1. Introduction

Since the 1970s, there has been a growing interest in the use of complementary and alternative medicine (CAM) around the world. CAM includes all medical and health care-related practices that are not considered part of Western medicine or allopathic medicine, such as acupuncture, moxibustion, and traditional Chinese medicine. The reasons for the developing interest in CAM are complex but likely include concerns over the rising price of health care, the growing wariness in the patient population towards the safety and efficacy of medications, and the limited effectiveness of biomedicine for the treatment of chronic diseases and diseases for which there are no findings upon performing the physical examination. Allopathic medicine has been highly successful up to this point in treating infectious diseases, which have been the majority of health concerns faced by patients until the 1970s. As people now begin to see the limitations of Western medicine, they are rediscovering traditional medicine in search of new solutions and treatments.

Japanese medical system is unique around the world because it is the only country where we can see the integrated use of modern and traditional medicine in daily practice. Today's Japanese physicians, who are all trained in allopathic medical schools, use biomedicine and traditional Japanese medicine (Kampo medicine) together in the clinic and even in the university hospital in combination with high tech medicine. Kampo medicine, which originates from ancient China, had been Japanese primary health care system for over 1500 years prior to the Meiji Restoration (1868–1912). It was not until the late 1700s, with the introduction of anatomy and surgery along with the successful use of the smallpox vaccine, that Western medicine began to take hold and develop into its own separate medical system in Japan. Both systems developed independently, although Kampo remained the system of choice until the late 1800s. In 1874, the government passed the Medical Care Law, which called for the adoption of the German model of health care and the abrogation of Kampo medicine. All Japanese physicians from that time onward were now only trained in allopathic medicine and Kampo practitioners were no longer considered legitimate medical professionals by the government. However, despite the government's attempts to eliminate Kampo medicine, early 20th century physicians continued to work towards reinstating Kampo as an official part of Japanese health care system. In 1967, the first four Kampo formulas were approved for coverage under the national health insurance system. As of today, this number has increased to 148 approved prescriptions. In 2001, the Ministry of Education, Science, and Technology also set new guidelines that called for the incorporation of Kampo medicine into the core curriculum of Japanese medical schools, and as of 2005, all medical schools in Japan have made the necessary changes to their curricula.

Today, Kampo is seen as a well-integrated component of Japanese medical system. Previous studies, such as those carried out by Fujiwara et al. [1], Yamashita et al. [2], and Watanabe et al. [3] that focus on the status of CAM in Japan have shown that up to 82% of Japanese physicians are familiar with the term “Kampo” and 78% use it in practice. This article evaluates the extent of Kampo's integration in daily practice. We predict that there has been an increase in the knowledge and usage of Kampo amongst physicians and that distribution and attitudes of Kampo differ among the different specialties. This article specifically provides the Kampo experience by physicians and a breakdown of Kampo usage among specialties. By looking at patterns in Kampo usage for each specialty, we hope to provide insight for improving Kampo practice in each field.

## 2. Methods

### 2.1. Sample and Data Collection

The survey of the usage of Kampo medicine by physicians was sponsored by the Japan Kampo Medicine Manufacturers Association and created by a third-party research group, TM Marketing Inc. An invitation to take the internet-based questionnaire was sent to 1,800 physicians randomly chosen from 17,164 physicians who agreed to be registered as a potential responder to the questionnaires by TM Marketing Inc., mostly non-Kampo experts who had never exposed to Kampo education, enrolled to TM Marketing Inc. which is a commercial contract research organization sponsored by Japan Kampo Medicine Manufactures Association. The study samples consisted of physicians practicing throughout Japan and the ratio of general physicians and hospital physicians reflects the ratio of Japan provided by the Ministry of Health, Labor, and Welfare of Japan (http://www.mhlw.go.jp/toukei/list/33-20.html). Total 684 physicians answered the questionnaires between August and September 2008.

Participants were first asked general profile questions, which included age, gender, and specialty. Secondly, the question was if they use Kampo medicine in daily practice and for those who responded affirmatively, additional Kampo-related questions were asked to get a sense of each participant's attitudes and beliefs towards Kampo. Questions, such as why s/he started to prescribe Kampo drugs and how long s/he has used Kampo in daily practice, the number of formulas s/he prescribes, and for which diseases Kampo is prescribed, were asked. The participants were also queried about their beliefs towards the reliability of Kampo diagnosis, the effectiveness of Kampo formulas, and the future of Kampo. Those physicians who responded not to use Kampo medicine in daily practice were asked for their reasons not to do so. To compare the indications for Kampo medicine in a university hospital, we searched the disorders of 1,691 new patients in the clinic of Center for Kampo Medicine in Keio University Hospital in 2005 and 2006.

## 3. Results

### 3.1. Participants Profile (Table 1)

Questionnaire participants were comprised mainly of physicians working in a hospital setting (72%), of which 12.9% work in university hospitals, 32.5% in teaching hospitals, and 26.6% in private nonteaching hospitals. The participants varied in age, with 19.6% (*n* = 134) of participants being less than 39 years of age and 5.8% (*n* = 40) over 60 years. The major age group was of 40–49 years (*n* = 310). 92.8% (*n* = 635) of participants were male, and 71.5% (*n* = 489) specialized in one of the following specialties: internal medicine (*n* = 232), surgery (*n* = 72), psychiatry (*n* = 56), orthopedics (*n* = 52), pediatrics (*n* = 47), or obstetrics/gynecology (OB/GYN) (*n* = 30).

### 3.2. Kampo in Daily Clinical Practice

**Figure 1:**
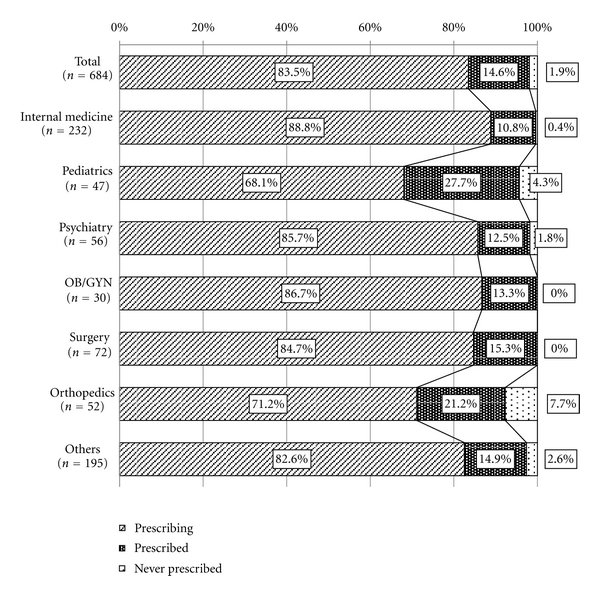
Physicians' experience of Kampo treatment. Total and each specialist's experiences of Kampo treatment. Oblique line bar shows the ratio of physicians who are prescribing Kampo drugs, vertical line bar, the ratio of physicians who have prescribed Kampo drugs before, dotted bar, the ratio of physicians who have never prescribed Kampo drugs.

Participating physicians were asked whether they use Kampo medicine in some capacity in their daily practice. Questionnaire results show that 83.5% of respondents (*n* = 571) use Kampo in their practice (Figure 1). Of the specialties surveyed, Kampo was most commonly used amongst internists (88.8%) and least commonly used amongst pediatricians (68.1%).

Table 2 shows the average number of Kampo prescriptions used by each specialty. Overall, 41.7% of physicians responded that they use 1–4 formulas and 30.1% use 5–9 formulas in the clinic. However, usage varied by specialty. For instance, 9.7% of internists and 7.7% of OB/GYN specialists responded that they use as much as 25 or more formulas in the clinic, whereas 73.0% of orthopedic surgeons, 54.1% of surgeons, 53.1% of pediatricians, and 45.8% of psychiatrists responded that they typically use only 1–4 formulas.

**Figure 2:**
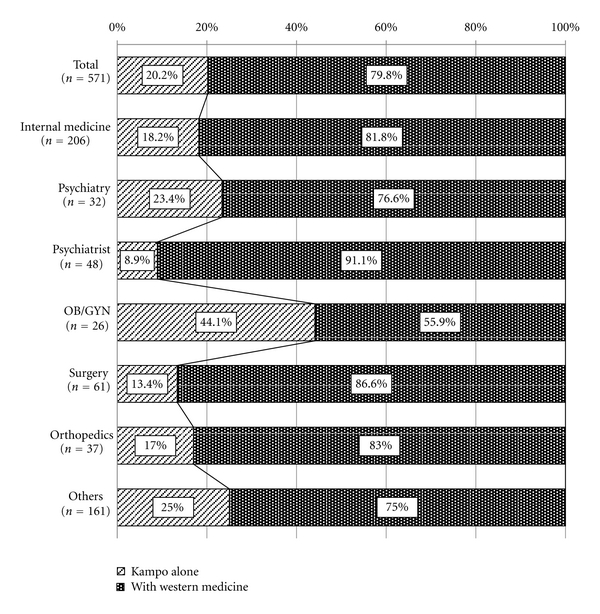
Treatment Style of usual practice of Kampo. Physicians who use Kampo in daily practice answered to the question whether they prescribe Kampo alone or together with Western medicine. Oblique line bar shows the ratio of physicians who prescribe Kampo drugs alone when physicians think that Kampo drug is necessary for treatment. Vertical line bar shows the ratio of physicians who prescribe Kampo drugs together with Western medicine.

Physicians were then asked about Kampo use for their patients (Figure 2): 79.8% responded that their patients use Kampo medicine along with Western medicine for treatment. The remaining 20.2% of physicians answered that their patients use Kampo medicine alone for treatment, although this also varied by specialty. As much as 44.1% of OB/GYN patients, for example, take Kampo medicine alone for treatment.

### 3.3. Current Attitudes toward Kampo Medicine

**Figure 3:**
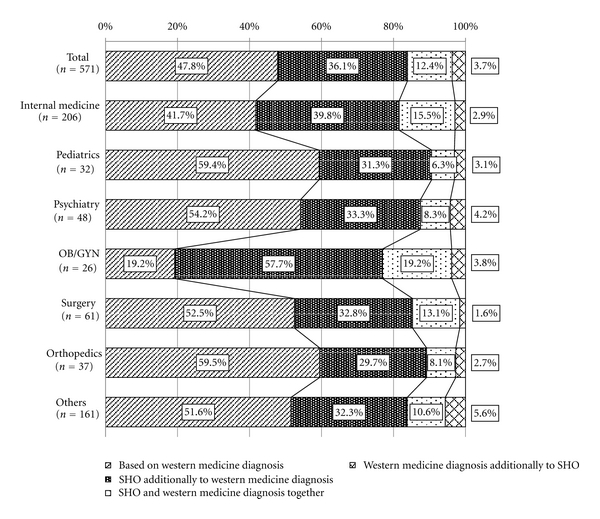
Selection of Kampo drugs. When Kampo using physicians prescribe Kampo drugs, based on what do they choose Kampo? Data is shown by ratio of total number of physicians and each specialist. Oblique line bar shows the ratio of physicians who prescribe Kampo drugs based on Western medicine diagnosis. Vertical line bar shows the ratio of physicians who prescribe Kampo drugs based on SHO (Kampo diagnosis) additionally to Western medicine diagnosis. Dotted bar shows the ratio of physicians who prescribe Kampo drugs based on SHO and Western medicine diagnosis together. Cross line bar shows the ratio of physicians who prescribe Kampo drugs based on Western medicine diagnosis additionally to SHO.

When asked about the importance of Kampo diagnosis (SHO), 47.8% of physicians overall replied that they relied mainly on Western diagnosis and 36.1% considered Kampo diagnosis (SHO) additionally to Western diagnosis (Figure 3). Only 12.4% of physicians gave both diagnosis with equal weight. In OB/GYN, only 19.2% answered that they give Western diagnosis only and 57.7% considered SHO additionally to Western diagnosis.

**Figure 4:**
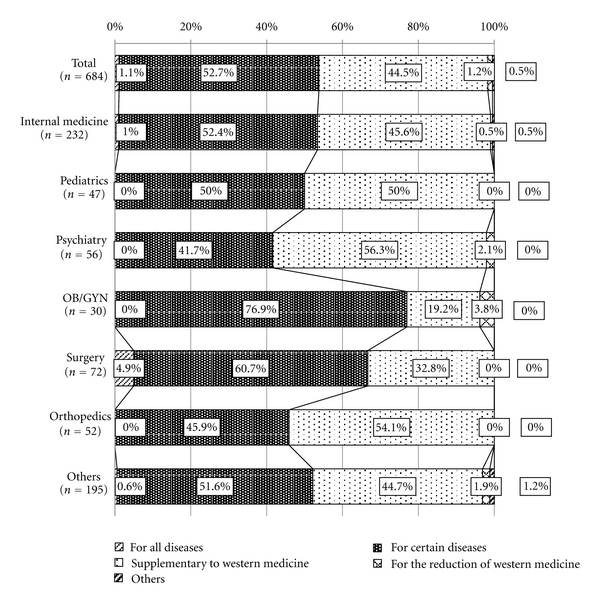
Indications for Kampo treatment. Oblique line bar shows the ratio of physicians who select Kampo for all diseases as a first choice. Vertical line bar shows the ratio of physicians who select Kampo treatment for certain diseases as a first choice. Dotted bar shows the ratio of physicians who select Kampo treatment as a supplement to Western medicine treatment. Cross line bar shows the ratio of physicians who use Kampo to reduce the side effects of Western medicine treatment. Right oblique line bar shows the others.

Physicians were also asked if they would choose Kampo as a first choice treatment for their patients (Figure 4). The majority of physicians (52.7%) responded that for certain conditions, they would prescribe Kampo as a first-line treatment, and other 44.5% replied that they use Kampo to complement Western medicine treatments. Interestingly, 4.9% of surgeons responded that they always offer Kampo as a first-choice treatment, and 76.9% of OB/GYN specialists replied that they would provide Kampo as the first-line of treatment for certain conditions.

**Figure 5:**
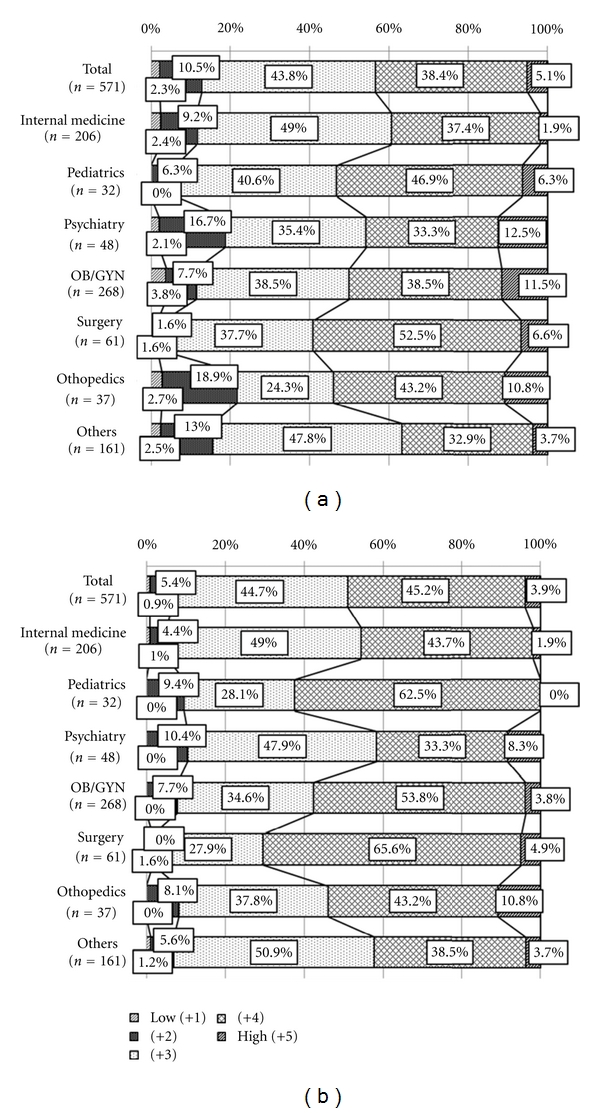
Satisfaction rate of Kampo treatment by (a) physicians and (b) patients. Satisfaction of Kampo treatment was rated from 1 (low) to 5 (high) by (a) physicians and (b) patients.

When asked to rank the satisfaction of Kampo treatment based on a 5-point scale, with a score of 5 being the highest ranking (Figure 5(a)), 43.5% of physicians gave a score of 4 or 5. This varied slightly across specialties: whereas only 39.3% of internists gave a score of 4 or 5, 59.1% of surgeons responded that Kampo was highly effective.

Additionally, physicians were asked about the patients' satisfaction (Figure 5(b)). Totally, 49.1% of physicians ranked patients' satisfaction in score 4 or 5, while 70.5% of surgeons gave a sore of 4 or 5.

Participating physicians were then asked the information resource of Kampo medicine and reasons for why they began to use Kampo in their practice (Table 3). Top information resource was from medical representatives of Kampo pharmaceutical company (45.7%), followed by other doctors (44.1%), issues or articles of medical journal (35.2%).

As for the incentive, the top reasons were (1) treatment with Western medicine alone was not sufficient for treating patients for certain conditions (56.4%) and (2) patients requested to take Kampo medicine (44.3%). 

Physicians who responded that they do not use Kampo medicine in daily practice (*n* = 113) were asked the reasons for not doing so (Table 4). Fifty physicians (44.2%) replied that they thought Kampo is difficult to use, 39.8% (*n* = 45) believed that there was not enough evidence currently available to support the use of Kampo in the clinic, and 32.7% (*n* = 37) answered that they believe that the effects of Kampo are too weak to be effective.

Table 5 shows the top 10 disorders for Kampo choice. Common cold was the top (46.8%) and followed by constipation (37.3%), muscle clamp (36.4%), menopausal syndrome (35.6%), and so forth. All of them were common disease and not life threatening. When this result was compared to the cases in the university hospital (Table 6), atopic dermatitis was the most common disease. Patients visit university hospital after western treatment with high expectations. As a fact, atopic dermatitis is the second most indication for Kampo treatment as cancer is ranked to number three in the university hospital. After surgery, cancer patients visited the Kampo clinic in expectation of prevention of further recurrence or metastasis. Additionally, patients rely on Kampo treatment for the palliative care.

### 3.4. Expectations toward Kampo's Future

**Figure 6:**
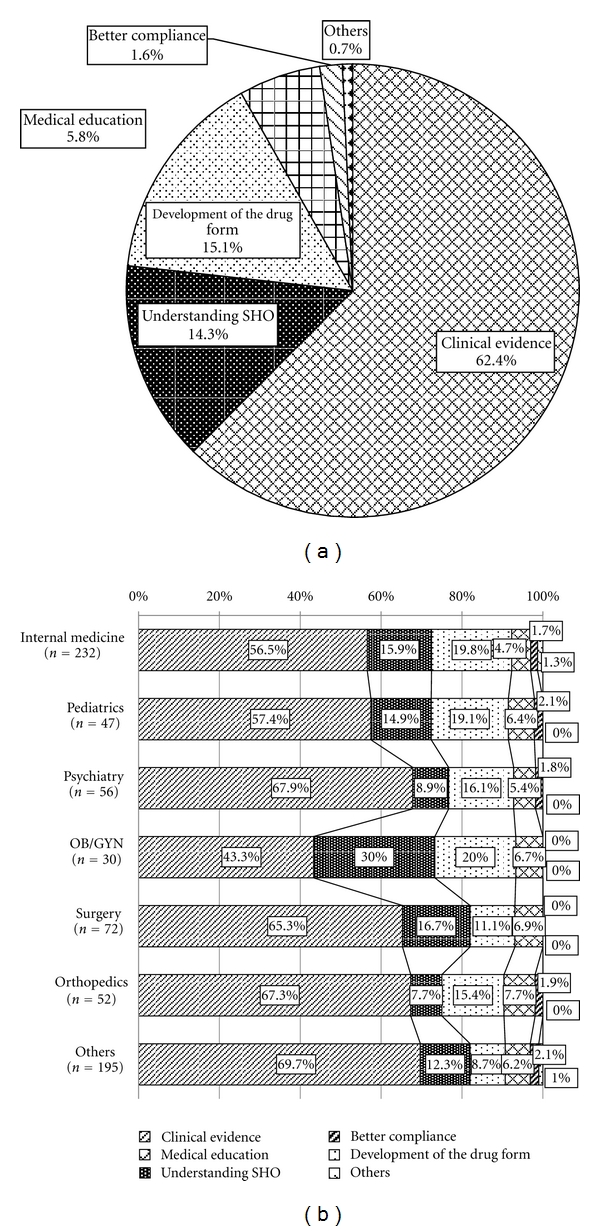
Expectation for Kampo in future. (a) Overall physicians' expectation. Cross oblique line pie shows the ratio of physicians who expect clinical evidence of Kampo treatment. Dense pie shows the physicians who expect the understanding of SHO in modern way. Dotted pie shows the ratio of physicians who expect the development of Kampo drug form (easier to take), grid pie shows the physicians who expect more Kampo education. Oblique line pie shows the ratio of physicians who expect better compliance of Kampo drugs (less intake or dose reduction). (b) Expectation for Kampo in each specialty.

The final section of the questionnaire asked all participants (*n* = 684) about their expectations for Kampo medicine in the future (Figure 6). The majority of physicians (62.4%) replied that more clinical evidence will be necessary before we can see an increase in the usage of Kampo in the clinic. Another 15.1% responded that the development of a Kampo drug form, which is easier to administer, would increase its usage amongst physicians, and 14.3% believed that it will be necessary to modernize Kampo diagnostic methods, in particular, develop a new understanding of the concept of *SHO* (a patient's individual diagnostic pattern), before Kampo can be completely integrated in Japanese current medical system. Others recommendations, such as changes in medical education (5.8%) and in better compliance (1.6%), were also made.

## 4. Discussion

### 4.1. Kampo Medicine Is Well Integrated in Japanese Current Medical System

With the passing of the first Medical Care Law in 1874, all systematic Kampo education was stopped and only Western medicine was offered at medical schools and up until the last forty years, this model had remained the basis for Japanese medical system. Today, we can see that Japanese physicians rely mainly on Western medicine for the diagnosis and treatment of patients. However, although Western medicine continues to be the principal standard of medical care, Kampo medicine has also gradually regained prominence in Japan's current medical system and the results of the study strongly suggest that Kampo medicine is now a well-integrated part of Japanese medical system. Today 83.5% of Japanese physicians use Kampo in daily practice, and of this set, 79.8% use Kampo along with Western medicine to treat patients. The other 20.2% of the participants stated that they would prescribe Kampo alone as the first-line choice of treatment. Compared to the results of previous studies, like that of Imanishi et al. [4] which found that 70% of the participating physicians use Kampo in their clinic, there has been an increase in the number of physicians who practice Kampo.

We speculate that there are several factors throughout Kampo's history that have allowed for its revival since the Meiji Restoration in 1867. A combination of physicians' efforts, advancements in science and research, and changes in government policy have all played some part in allowing for Kampo's integration into Japanese current medical system. Physicians' efforts to keep Kampo alive while government attempted to suppress it played a key role in Kampo's survival into contemporary times. In 1934, Dr. Domei Yakazu and Dr. Keisetsu Otsuka, two prominent physicians of their generation, formed the Japanese Society of Kampo Medicine. Through this association, they created a Kampo training program and teaching materials, such as a Japanese translation of the *Shan hang lun,* and it helped to spread knowledge of Kampo to other physicians. This society would eventually lead the way to the rise of the Japan Society for Oriental Medicine, which to this day continues to promote Kampo medicine through educational programs, academic meetings, and through the publication of its research journal. Other than the establishment of physician associations, in 1936, Dr. Yakazu and Dr. Otsuka also set up the first course on Kampo Medicine at Takushoku University. Later in 1972, Dr. Otsuka would also go on to establish the Oriental Medicine Research Center at Kitasato Institute, an institution open to all interested in studying Kampo medicine including international students.

 At the same time, advancements in science and research also played a role in allowing for the integration of Kampo into the current medical system. By 1957, pharmaceutical companies developed a methodology for mass-producing standardized Kampo formulas in spray-dry extract form. Kampo medicine could now be distributed quickly around the country and sold as an over-the-counter medication in drug stores. The development of technology, such as photodioide array detectors and high-performance liquid chromatography (HPLC), also allowed for the rapid expansion of Kampo research. HPLC analysis, which allows us to examine how the different components of a Kampo formula interact with one another, is a powerful method for understanding the molecular mechanisms by which Kampo formulas act. Due to this method we now understand, for example, the mechanisms of commonly used formulas like rikkunshito [5–9] and juzentaihoto [10–13].

Changes in the government policy have also allowed for Kampo's integration with modern Japanese medicine. In 1967, the government accepted the first four Kampo formulas for coverage under the national health insurance system. By 1976, the number of approved formulas covered by insurance increased from four to forty-one. This change in the government policy caused an exponential increase in Kampo usage all over the country. Now that Kampo was covered under national insurance, physicians were more willing to prescribe Kampo formulas to patients. Physicians now also had more incentive to learn how to effectively use Kampo medicine as the government has sanctioned it as a legitimate treatment alternative. The pharmaceutical industry responded to the change in government policy by increasing their efforts in research and development of Kampo products. Between 1976 and 1992, the market sales of Kampo medicine grew therefore 10-fold. Further, the government also established the Good Manufacturing Practice (GMP) law in 1987 to ensure that all Kampo products are uniform and of high quality. Furthermore, in 2001, the Ministry of Education, Science and Technology set the first guidelines for including Kampo education in the core curriculum for medical schools around the nation. A systematic system for Kampo education was seen as necessary for ensuring that the future generations of Japanese physicians would be able to integrate Kampo in their practice as well. All together, physician efforts, advances in Kampo research and science, and changes in the government policy have all allowed for Kampo's integration into the Japanese medical system.

### 4.2. Differences in Kampo Usage by Specialty

Although 83.5% of Japanese physicians overall use Kampo to some capacity in daily practice, there are clearly differences in usage amongst the different specialties. From the results, we saw that certain aspects of Kampo medicine, such as the value different specialists placed on Kampo diagnosis or the percentage of patients to whom they prescribe Kampo formulas, varied greatly depending on the specialty. We attribute these differences to the nature of the conditions commonly seen by each specialty as well as to the history of Kampo use in each.

The most mentionable differences in Kampo usage was within the OB/GYN specialists. Overall, we see higher usage and a more positive perception of Kampo in this specialty. 57.7% of OB/GYN specialists answered that they give equal consideration to Western and Kampo diagnoses and 44.1% responded that they give Kampo medicine alone as treatment for their patients. Furthermore, amongst all the specialties, OB/GYN has the highest percentage of physicians (76.9%) who have used Kampo for more than 16 years in daily practice (data not shown). The likely reason for the extensive use and support for Kampo in OB/GYN is Kampo's long history for treating women's disorders. Women suffering from conditions such as premenstrual syndrome usually do not present with any findings upon physical examination. The symptoms experienced by these women are often subjective in nature, making it difficult for physicians to use Western medicine for treatment. As a result, historically Japanese physicians have preferred to use Kampo, and pharmaceutical companies and research institutions have focused their research and development of Kampo formulas with this in mind. For example, when the Kampo-drug manufacturer Tsumura & Co. was first established in 1893, the company's first product was for the treatment of women's disorders. Since Kampo has a well-established reputation for effectiveness in treating women's disorders, OB/GYN specialists are more inclined to using it in daily practice.

Kampo usage is also unique amongst surgeons. Like with OB/GYN the nature of the conditions seen by surgeons and the history of Kampo usage in the field of surgery have influenced the way present-day Japanese surgeons use Kampo. The data show that 54.1% of surgeons use only a limited number of formulas, that is, one to four, to treat patients. The limited number of Kampo formulas used in this field is probably due to the fact that Kampo is used primarily for wound care during surgery and for the reduction of swelling from fractures. At Keio University hospital, 100% of surgical patients also take Daikenchuto extract, a Kampo medicine, to accelerate postoperative recovery [14, 15]. The results also show that surgeons tend to be more supportive of Kampo's use: 70.5% ranked Kampo as highly effective with a score of 4 or 5 (Figure 5(a)), and 4.9% responded that they always offer Kampo as a first-choice treatment as opposed to a treatment as supplement to Western medicine (Figure 4). We can only speculate the reasons for why such a positive perception of Kampo exists in the field of surgery. One reason may be that Kampo has been used in surgery since the field was introduced in Japan in the late 1700s: as early as 1805, Hanaoka Seishu resected a breast cancer using Kampo as the world's first general anesthesia. As with OB/GYN, since Kampo has a well-established reputation in the field of surgery, so present-day surgeons are more likely to use Kampo in daily practice.

Within internal medicine, we see yet a different pattern of Kampo usage in the clinic. Amongst all the specialties, internal medicine had the highest number of physicians (88.8%) who responded that they use Kampo daily in practice (Figure 1), and 9.7% of this group replied that they commonly prescribe 25 or more Kampo formulas (Table 2). We attribute the high rate of usage and diversity of formulas used to the fact that internists see the widest variety of patients. Moreover, in February 1998, the Japanese Society for Oriental Medicine established Kampo Medicine as a specialty that physicians can enter after board certification. These specialists often complete their training in internal medicine before choosing to specialize in Kampo. Thus, our sample group of internists likely includes physicians who have chosen to specialize in Kampo. Since Kampo specialists must know all 148 Kampo prescriptions covered by the Japanese health insurance, they can use a wider array of Kampo formulas. This may be another reason why we see a high rate of usage and diversity of formulas used amongst internists.

Based on the results of the questionnaire, Kampo medicine seems less popular as a treatment option in pediatrics, psychiatry, and orthopedics as compared to OB/GYN, surgery, and internal medicine. 59.4% of pediatricians, 54.2% of psychiatrists, and 59.5% of orthopedic surgeons responded that they rely only on Western diagnosis for treating patients (Figure 3). In addition, the majority of physicians in these specialties who use Kampo use only 1–4 different types of formulas (Table 2). We can only speculate reasons why these trends exist. Perhaps current Western treatments are sufficient enough in these specialties that physicians do not need to look into alternative treatments for patients. Furthermore, whereas in OB/GYN or surgery there has been a long tradition of using Kampo for certain conditions, the same does not seem to exist for pediatrics, psychiatry, and orthopedic surgery. Thus, specialists in these fields may know less about potential Kampo treatments for their patients. Another reason is probably the lack of clinical evidence on the efficacy of Kampo formulas. Pediatricians, for example, work with a special population of patients. They may be more hesitant to use Kampo for fear of unknown side effects in children. Pediatricians must also take the parents' wishes into consideration: parents may not want their children to take herbal medicine if a Western treatment is available. Children also often refuse to take bitter-tasting medicine, making it difficult to prescribe certain undelightful tasting Kampo formulas. However, in this case, the combined use with flavoured jellies could help to cover the bitterness of these medications. The same reasoning may be applied to psychiatry and orthopedic surgery. In psychiatry, there has only been limited evidence on the effectiveness of combining Kampo with antipsychotics for patients with schizophrenia. However, the amount of clinical evidence supporting the use of Kampo in psychiatry is still scarce, making psychiatrists cautious about prescribing Kampo to their patients. Furthermore, as with pediatrics, psychiatrists may be hesitant to prescribe Kampo because of side effects. One such example is with *ginkgo*, an herb used in formulas against dementia. In combination with aspirin, NSAIDs, anticoagulants, or other platelet inhibitors, it has been shown to significantly increase the bleeding tendency of the patient [16–20]. For these specialties, more clinical data are necessary before we might see an increase of usage in these fields.

### 4.3. Toward the Modernization of Kampo Medicine

Of the 684 participants, 113 replied that they do not use Kampo medicine in daily practice. In this subset, 44.2% (*n* = 50) stated that they found Kampo difficult to use, 39.8% (*n* = 45) believed that there was not enough evidence currently available to support the use of Kampo in the clinic, and 32.7% (*n* = 37) answered that they believe the effects of Kampo are weak. Questionnaire participants also suggested that changes in medical education and in Kampo prescription would be beneficial for the spread of Kampo use amongst Japanese physicians. The need for clinical evidence, for improvements in medical education, and for improvements in Kampo prescription have all have been long-standing issues Kampo has faced.

Even in 1976 when the government had approved an additional 37 formulas to be covered under the national insurance system, members of the government hesitated because of the lack of clinical evidence on the effectiveness and efficacy of Kampo formulas, despite data collected by pharmaceutical companies and research institutions on the mechanistic action of many Kampo formulas. At present, it is still difficult for researchers to design randomized controlled trials (RCTs) given the individualized nature of Kampo treatments. Randomized clinical trials, which are the best design for testing the efficacy or effectiveness of a drug, require standardization of treatment across the patient population. This is difficult to achieve with Kampo since Kampo treatments are individualized according to a patient's *sho* pattern and diagnosis. Keio University is currently developing a data-mining system that collects individual patient data for analysis to overcome this challenge [21].

The need for comprehensive Kampo education has been another problem that has limited the widespread use of Kampo in clinics. As physicians have indicated in the questionnaire, many feel that present-day physicians, who do not use Kampo in the clinic, do not use Kampo because they do not have a comprehensive understanding of Kampo diagnosis or of how to use Kampo formulas effectively (Figure 6). Unfortunately this remains as a residual effect of the Meiji government's suppression of systematic Kampo education. Although individual physician efforts were enough to sustain interest of Kampo in the medical community and allow for its revival, the modern form of Kampo that was taught placed less emphasis on the theory behind Kampo. The change in government policy in 2001, which mandated the incorporation of Kampo education in medical school curriculums, is one step toward resolving this issue. Physician associations such as the Japan Society for Oriental Medicine also provide educational programs for interested physicians. As more physicians are exposed to Kampo medicine at an earlier stage in their career, this will hopefully provide more opportunity for these future generations of physicians to develop a better understanding of *sho* diagnosis and the use of Kampo formulas.

Finally, physicians believe that changes in the formula form and prescription protocol of Kampo formulas would help to increase usage of Kampo medicine in clinics. Most Kampo formulas are currently taken in powder form. A typical prescription requires patients to take these powder formulas with hot water three times a day for an extended period of time (weeks to months). Many physicians have found the powder form difficult to administer and due to the frequency it needs to be taken, patients might skip some intakes and this may be why some believe the effects of Kampo to be weak. Moreover, Japanese physicians believe that the current prescription protocol is difficult for patients to adhere because of the frequency and duration for which patients must take the Kampo medicine. If changes were made to the formula form and prescription protocol of Kampo medicine to make its appliance more easy, it is possible that this will influence more physicians to prescribe Kampo in the clinic.

### 4.4. Physicians' Attitude to CAM in Other Countries

The field of integrative medicine and holistic healthcare has grown largely in the world. There have been many articles on the use of complementary and alternative medicine (CAM) from nation's point of view. Eisenberg et al. [22] reported that Americans were spending more in aggregate on CAM products and services than they were on visits to conventional primary care doctors. According to a 2004 survey from the Centers for Disease Control and Prevention, 74.6% of U.S. adults acknowledge using some form of complementary and alternative medicine at some point in their lives and 62% had used CAM services in the previous 12 months [23]. Commonly used CAM therapies were nonvitamin, nonmineral, natural products (17.7%), deep breathing exercises (12.7%), meditation (9.4%), chiropractic or osteopathic manipulation (8.6%), massage (8.3%), and yoga (6.1%). Estimated population of traditional botanical therapy users was only 0.02% of whole nations. This means people got the nonvitamin, nonmineral, natural products mainly by themselves and did not visit traditional medicine experts.

In US, CAM is practiced mainly by nonphysician health care practitioner and the collaboration is emphasized [24]. This is because most CAM services are not covered by insurance plans [25]. Also in terms of education, residents and medical students are taught to understand how to refer patients to other CAM providers [26].

 The situation is different in Germany. In Germany, the overall percentage of individuals with experience in CAM increased from 52% in 1970 to 73% in 2002 [27]. National survey of German office-based physicians in outpatients care (GPs and specialists) authorized by the National Association of Statutory Health Insurance showed many modalities of CAM including acupuncture (37%) and traditional Chinese medicine (TCM) are used by physicians [28]. Another article focusing on family practice physicians in Germany showed that neural therapy, phytotherapy, and acupuncture were the most commonly used therapy [29].

 In Japan, among CAM modalities, only Kampo medicine is taught to prescribe in medical schools and other CAM therapies are not educated. This bias is based on the culture background on the long medical history in Japan. Additionally, only Kampo and very limited indications of acupuncture are covered by national health insurance system. Also in terms of the quality management [30], Kampo products are well controlled and quite safe. The quality control of the purity as well as toxicity is standardized in Japan, following the Japanese pharmacopoeia and internationally established regulations for Good Manufacturing Practice (GMP) and Good Laboratory Practice (GLP). The standardization of the main components has become possible and this is a precondition of clinical research [21]. In future, it is possible that other CAM modalities will be demanded and used by physicians if the nation demands it and covered by insurance.

## 5. Conclusion

Despite recent advances in Kampo's status in the current medical system in Japan, Kampo medicine must still modernize before we see more Japanese physicians use it in their practice. The two main issues that Kampo currently faces are (1) the need for clinical evidence to support its efficacy; (2) improvement of clinical training and Kampo education programs. Although Kampo is widely used today, the majority of physicians (62.4%) believe that clinical evidence will be necessary before more physicians can support the use of Kampo in the clinic. This would be of particularly high impact for those specialties, such as psychiatry or orthopedics, which do not have a history of Kampo use for their specialties. Improvements in Kampo medical education would help Kampo gain more ground amongst physicians. 44.2% of physicians who currently do not use Kampo in the clinic responded that they felt that Kampo was difficult to use. If improvements to Kampo medical education are made such that medical professionals can begin to practice Kampo diagnosis and treatment at an earlier point in their careers, Kampo can be better integrated in their practice in the future.

## Figures and Tables

**Table 1:** Profile of the physicians of this study.

	Total (*n* = 684)	Internal medicine (*n* = 232)	Pediatrics (*n* = 47)	Psychiatry (*n* = 56)	OB/GYN (*n* = 30)	Surgery (*n* = 72)	Orthopedics (*n* = 52)	Others (*n* = 195)
Age (y/o)								
~39	134 (19.6%)	43 (15.9%)	10 (21.3%)	16 (28.6%)	4 (13.3%)	11 (15.3%)	7 (13.5%)	49 (25.1%)
40~49	310 (45.3%)	100 (43.1%)	15 (31.9%)	15 (26.8%)	15 (50.0%)	41 (56.9%)	31 (59.6%)	93 (47.7%)
50~59	200 (29.2%)	78 (33.6%)	18 (38.3%)	21 (37.5%)	7 (23.3%)	18 (25.0%)	11 (21.2%)	47 (24.1%)
60~	40 (5.8%)	17 (7.3%)	4 (8.5%)	4 (7.1%)	4 (13.3%)	2 (2.8%)	3 (5.8%)	6 (3.1%)
Sex								
Male	635 (92.8%)	212 (91.4%)	40 (85.1%)	50 (89.3%)	27 (90.0%)	70 (100%)	52 (100%)	182 (93.3%)
Female	49 (7.2%)	20 (8.6%)	7 (14.9%)	6 (10.7%)	3 (10.0%)	0 (0%)	0 (0%)	13 (6.7%)
Clinical settings								
University hospital	88 (12.9%)	12 (5.2%)	8 (17.0%)	6 (10.7%)	2 (6.7%)	16 (22.2%)	5 (9.6%)	39 (20.0%)
Educational hospital	222 (32.5%)	83 (35.8%)	24 (51.1%)	20 (35.7%)	14 (46.6%)	24 (33.4%)	29 (55.8%)	17 (8.6%)
Noneducational hospital	183(26.6%)	83(35.8%)	5(10.7%)	20(35.7%)	6(20.0%)	21(29.1%)	10(19.2%)	37(19.0%)
Clinic	182 (26.2%)	96 (41.4%)	10 (21.3%)	7 (12.5%)	8 (26.7%)	8 (11.1%)	7 (13.5%)	43 (22.1%)
Others	13 (1.9%)	5 (2.2%)	0 (0%)	3 (5.4%)	0 (0%)	3 (4.2%)	1 (1.9%)	1 (0.5%)

**Table 2:** The number of Kampo medicine used for patients.

Number of Kampo drugs	Total (*n* = 571)	Internal medicine (*n* = 206)	Pediatrics (*n* = 32)	Psychiatry (*n* = 48)	OB/GYN (*n* = 26)	Surgery (*n* = 61)	Orthopedic (*n* = 37)	Others (*n* = 161)
1~4	238 (41.7%)	57 (27.7%)	17 (53.1%)	22 (45.8%)	3 (11.5%)	33 (54.1%)	27 (73.0%)	79 (49.1%)
5~9	172 (30.1%)	66 (32.0%)	10 (31.3%)	15 (31.3%)	11 (42.3%)	15 (24.6%)	4 (10.8%)	51 (31.7%)
10~14	90 (15.8%)	43 (20.9%)	1 (3.1%)	8 (16.7%)	5 (19.2%)	9 (14.8%)	4 (10.8%)	20 (12.4%)
15~19	20 (3.5%)	9 (4.4%)	1 (3.1%)	0 (0%)	3 (11.5%)	2 (3.3%)	2 (5.4%)	2 (1.9%)
20~24	22 (3.9%)	11 (5.3%)	2 (6.3%)	2 (4.2%)	2 (7.7%)	1 (1.6%)	0 (0%)	4 (2.5%)
over 25	29 (5.1%)	20 (9.7%)	1 (3.1%)	1 (2.1%)	2 (7.7%)	1 (1.6%)	0 (0%)	4 (2.5%)

Average number	7.8	10.4	7.3	6.0	11.3	5.6	4.0	6.2

**Table 3:** Information and reason to have started Kampo treatment (plural answers).

Information resource	( *n* = 571)
Medical representatives	261 (45.7%)
Recommendation from other doctors	252 (44.1%)
Issues or articles of medical journal	201 (35.2%)
Academic meetings	185 (32.4%)
Request from patients	176 (30.8%)
Seminar by pharmaceutical company	123 (21.5%)
Hospital seminar	50 (8.8%)
Internet	43 (7.5%)
Others	16 (2.8%)
Incentive	
More effective than Western medicine	324 (56.4%)
Request from patients	253 (44.3%)
Evidence reported in academic meeting	192 (33.6%)
Limitation of Western medicine	181 (31.7%)
Comprehensive therapy in Kampo	91 (15.9%)
Medical cost reduction	11 (1.9%)
Others	28 (4.9%)

**Table 4:** Reasons not to prescribe Kampo drugs (plural answers).

	Total (*n* = 113)	Internal medicine (*n* = 26)	Pediatrics (*n* = 15)	Psychiatry (*n* = 8)	OB/GYN (*n* = 4)	Surgery (*n* = 11)	Orthopdic (*n* = 15)	Others (*n* = 34)
Difficult to use	50 (44.2%)	10 (38.5%)	7 (46.7%)	3 (37.5%)	1 (25.0%)	5 (45.5%)	4 (26.7%)	20 (58.8%)
Short of clinical evidences	45 (39.8%)	10 (38.5%)	7 (46.7%)	2 (25.0%)	0 (0%)	5 (45.5%)	7 (46.7%)	14 (41.2%)
Not enough effectiveness	37 (32.7%)	13 (53.8%)	3 (20.0%)	2 (25.0%)	0 (0%)	3 (27.3%)	6 (40.0%)	9 (26.5%)
Patient's unpreferance	24 (21.2%)	3 (11.5%)	8 (53.3%)	2 (25.0%)	1 (25.0%)	4 (36.4%)	0 (0%)	2 (5.9%)
Only Western medicine is satisfactory	24 (21.2%)	10 (38.5%)	1 (6.7%)	2 (25.0%)	2 (50.0%)	4 (36.4%)	3 (20.0%)	2 (5.9%)
Others	13 (11.5%)	3 (11.5%)	3 (20.0%)	1 (12.5%)	0 (0%)	1 (9.1%)	1 (6.7%)	4 (11.8%)

**Table 5:** Top 10 disorders for Kampo choice.

	Disease name	Treated patients (%)
1	Common cold	46.8
2	Constipation	37.3
3	Muscle clamp	36.4
4	Menopausal syndrome	35.6
5	Bronchitis	34.2
6	gastritis	29.4
7	fatigue	25.4
8	ileus	18.0
9	edema	12.1
10	neurosis	12.1

**Table 6:** Top 10 disorders in a university hospital (Keio University Hospital) 1691 new patients in Keio University Hospital in 2005 and 2006.

	Disease name	Treated patients (%)
1	Atopic dermatitis	169 (9.0 %)
2	Cold sensation	117 (6.9 %)
3	Cancer therapy	92 (5.4 %)
4	Irregular menstruation	89 (5.3 %)
5	Dysmenorrhea	84 (4.9 %)
6	Dermatitis and eczema	61 (3.6 %)
7	Fatigue	47 (2.8 %)
8	Rhinitis	46 (2.8 %)
9	Insomnia	43 (2.5 %)
10	Infertility	40 (2.4 %)
